# Progression of Cerebellar Atrophy in Spinocerebellar Ataxia Type 2 Gene Carriers: A Longitudinal MRI Study in Preclinical and Early Disease Stages

**DOI:** 10.3389/fneur.2020.616419

**Published:** 2020-12-15

**Authors:** Anna Nigri, Lidia Sarro, Alessia Mongelli, Chiara Pinardi, Luca Porcu, Anna Castaldo, Stefania Ferraro, Marina Grisoli, Maria Grazia Bruzzone, Cinzia Gellera, Franco Taroni, Caterina Mariotti, Lorenzo Nanetti

**Affiliations:** ^1^Department of Neuroradiology, Fondazione Istituto di Ricerca e Cura a Carattere Scientifico (IRCCS) Istituto Neurologico Carlo Besta, Milan, Italy; ^2^Department of Medical Genetics and Neurogenetics, Fondazione Istituto di Ricerca e Cura a Carattere Scientifico (IRCCS) Istituto Neurologico Carlo Besta, Milan, Italy; ^3^Ospedale Martini, Turin, Italy; ^4^Laboratory of Methodology for Clinical Research, Oncology Department, Istituto di Ricerche Farmacologiche Mario Negri Istituto di Ricerca e Cura a Carattere Scientifico (IRCCS), Milan, Italy

**Keywords:** pre-symptomatic gene carriers, spinocerebellar ataxia (SCA2), cerebellar lobule segmentation, cortical thickness, cognitive assessment, cerebellar structural MRI, symbol digit modalities test

## Abstract

Spinocerebellar ataxias type 2 (SCA2) is an autosomal dominant inherited disease caused by expanded trinucleotide repeats (≥32 CAG) within the coding region of *ATXN2* gene. Age of disease onset primarily depends on the length of the expanded region. The majority of subjects carrying the mutation remain free of clinical signs for few decades (“pre-symptomatic” stage), but in proximity of disease onset subtle neurophysiological, cognitive, and structural brain imaging changes may occur. Aims of the present study are to determine the time-window in which early clinical and neurodegenerative MRI changes may be identified, and to evaluate the rate of the disease progression in both preclinical and early disease phases. We performed a 1-year longitudinal study in 42 subjects: 14 SCA2 patients (mean age 39 years, disease duration 7 years, SARA score 9 points), 13 presymptomatic SCA2 subjects (preSCA2, mean age 39 years, expected time to disease onset 16 years), and 15 gene-negative healthy controls (mean age 33 years). All participants underwent genetic test, neurological examination, cognitive tests, and brain MRI. Evaluations were repeated at 1-year interval. Baseline MRI evaluations in SCA2 patients showed significant atrophy in cerebellum, brainstem, basal ganglia and cortex compared to controls, while preSCA2 subjects had isolated volume loss in the pons, and cortical thinning in specific frontal and parietal areas, namely rostral-middle-frontal and precuneus. One-year longitudinal follow-up demonstrated, in SCA2 patients, volume reduction in cerebellum, pons, superior cerebellar peduncles, and midbrain, and only in the cerebellum in preSCA2 subjects. No progression in clinical or cognitive measures was observed in preSCA2 subjects. The rate of volume loss in the cerebellum and subcortical regions greatly differed between patients and preSCA2. In conclusion, our pilot study demonstrated that MRI measures are highly sensitive to identify longitudinal structural changes in SCA2 patients, and in preSCA2 up to a decade before expected disease onset. These findings may contribute in the understanding of early neurodegenerative processes and may be useful in future therapeutical trials.

## Introduction

Spinocerebellar ataxia type 2 (SCA2, OMIM 183090) is an autosomal dominant inherited disease caused by trinucleotide repeat expansion in the coding region of *ATXN2* gene. SCA2 is classified among the group of polyglutamine disorders (1), and accounts for ~10–25% of dominant familial cases. SCA2 is particularly frequent in Cuba, Mexico, Korea, India, Spain, and Italy (2). The usual clinical presentation is a progressive adult onset cerebellar syndrome associated with saccadic slowing, axonal sensory neuropathy, autonomic dysfunctions, motor neuron involvement, and cognitive abnormalities (1). Less frequently SCA2 patients may present a dopa-responsive Parkinsonism (3).

As in other polyglutamine diseases, the age at onset is inversely related to the number of triplet repeats, and the subjects carrying the mutation may remain free of symptoms and clinical signs for a few decades of their life (“asymptomatic or presymptomatic” stage). Clinical disease onset is characterized by the first appearance of gait difficulties or other obvious cerebellar features (“ataxic” stage). Between the asymptomatic and the overt ataxic stage, gene mutation carriers experience a “preclinical or prodromal” stage in which unspecific neurological symptoms are recognized, and early neurophysiological, cognitive, and structural brain imaging changes may be measured (4–7). In particular voxel-based morphometry was shown to represent a potential source of biomarkers for disease severity and progression (8, 9). Neuroimaging studies in SCA2 demonstrated gray matter (GM) volume loss in the cerebellar hemispheres and vermis, brainstem, and thalamus, and also in the inferior frontal, parietal, and mesial temporal structures, as compared to controls (10–12). White matter damage was also observed in the corticospinal tracts, superior and inferior longitudinal, uncinated, and inferior occipital fasciculi, besides the cerebellar and brainstem regions compared to controls (13, 14). Gray matter loss in brainstem and cerebellum, particularly lobules V and VI, and in frontal cortex in SCA2 are already detectable in prodromal disease stages (4, 15–17).

At present only a few studies have evaluated preclinical SCA2 subjects (4, 5, 16–18). There is the need for longitudinal clinical and neuroimaging data for the implementation of future clinical trials on disease-modifying interventions (19). In this study, we prospectively followed a cohort of preclinical and early symptomatic SCA2 mutation carriers with the aims of establishing (i) the rate of the disease progression in preclinical and early disease phases; (ii) the time-window in which structural brain changes can be observed; and (iii) the correlations between clinical and neuroimaging outcome measures.

## Subjects and Methods

### Participant Groups

Between February 2015 and March 2017, we screened 44 adult subjects belonging to SCA2 diagnosed families. Fifteen individuals had overt ataxic signs and confirmed SCA2 genetic diagnosis, 14 were asymptomatic gene-mutations carriers (preSCA2) and 15 were gene-negative healthy controls (CTR). All participants were over 18 years and gave their written informed consent before undergoing study procedures. The study was approved by the local Ethic Committee.

Trinucleotide CAG repeated region in *ATXN2* (SCA2, MIM 183090) was analyzed as previously described (20). Participants who did not present ataxic symptoms were informed to have 50% risk of carrying the SCA2 expansion, and they had the choice to know about their genetic status in agreement with the genetic counseling protocol for predictive genetic test.

Presence and severity of ataxia was evaluated using the Scale for Assessment and Rating of Ataxia (SARA) (21). SARA score was used to differentiate patients (score ≥3) from asymptomatic subjects (score <3) (17). To assess neurological signs other than ataxia, we used the inventory of non-ataxia signs (INAS) (22).

Cognitive assessment was performed using a battery of cognitive tests including: Mini Mental State Examination Test (MMSE), digit span forward, copy and delayed recall of the Rey-Osterrieth complex figure (ROCF), phonemic (letters F-P-L) and semantic fluency tests (animals-fruits-car brands), and Symbol Digit Modalities Test (SDMT) (23–25). Scores were corrected for age, sex and education, according to the normative data. Disease duration was calculated based on subject's age at enrolment and age of ataxia manifestation. For preSCA2, age of expected disease onset was estimated according to the model described by Tezenas du Montcel et al. (26).

Exclusion criteria were the presence of psychiatric diseases, neurologic abnormalities others than spinocerebellar ataxia, claustrophobia, substance abuse, hydrocephalus, traumatic brain injury or intracranial mass. CTR subjects, SCA2 patients, and 9 out of 13 preSCA2 subjects repeated clinical evaluations and brain MRI at 1-year interval (±2 months).

### Structural MRI (sMRI) Acquisition and Analyses

Participants underwent MRI acquisition on 3T scanner (Achieva, Philips Healthcare NL) equipped with a 32-channel head coil. The MRI protocol included a high-resolution 3D T1-weighted (TR = 9.781 ms, TE = 4.6 ms, FOV = 240 × 240 mm, no gap, voxel size = 1 × 1 × 1 mm, flip angle = 8°, 185 sagittal slices), axial T2-weighted turbo spin echo, and 3D fluid attenuated inversion recovery sequences. Images were examined by neuroradiologists for standard diagnostic purposes and to exclude incidental findings in the study participants.

A region of interest (ROI) analysis on volume and thickness for cerebellum, on volume for subcortical regions, and on thickness for cortical regions, was conducted at baseline and follow up 3D T1-weighted images.

For cerebellum assessment, a pre-processing including a de-noising of the images, an intensity inhomogeneity correction, and a cropping step to limit the processing to the cerebellum was performed and a cerebellum lobule segmentation was obtained using CERES software (27) (see Supplementary Material). Volume and mean cortical thickness for each cerebellar lobule and for the whole cerebellum were extracted.

For evaluation of cerebral cortex, subcortical nuclei (i.e., bilateral putamen, caudate, pallidum, thalamus), corpus callosum, and brainstem substructures (i.e., medulla oblongata, pons, midbrain, and superior cerebellar peduncles), FreeSurfer software (http://freesurfer.net, version 6) was used (28, 29). White matter and pial surface were generated and manually corrected by an expert operator (C.P.) when errors where identified. Cortical thickness was calculated as the distance between the vertices of white and pial surface. The segmentations were performed according to Fischl et al. (30) for subcortical nuclei and corpus callosum, and according to Iglesias et al. (28) for brainstem (see Supplementary Material for extensive description). The volume of each segmented subcortical region was obtained. Due to no hypothesis on atrophy lateralization, we computed the mean of the left and right measures for the bilateral cerebellar lobules and subcortical nuclei. For the total volume of the corpus callosum, we summed the volumes of genu, body, and splenium segments.

For cortical regions, we assumed that the possible atrophy in preSCA2 subjects begins in the same regions where the atrophy is detectable in SCA2 patients. To this aim, a vertex-wise analysis using a General Linear Model approach was conducted to detect significant cortical thinning in SCA2 in comparison to CTR. To correct for multiple comparisons a Monte Carlo simulation was applied (cluster threshold: *p* < 0.01). Significant clusters of cortical thinning in the comparison SCA2 were neuroanatomically labeled based on the Desikan-Killiany cortical atlas and identified as cortical ROIs. Mean cortical thickness for each cluster (i.e., cortical ROI) was extracted for each participant (CTR, preSCA2, and SCA2) at baseline and follow-up evaluation. All extracted volumes (i.e., whole cerebellum, lobules of cerebellum, and subcortical regions) were expressed as percentage of total intracranial volume (TIV), to account for inter-subject variability (31).

### Statistical Methods

Baseline clinical (SARA score, total INAS count, cognitive scores) and sMRI quantitative data (i.e., volumes of the subcortical regions, mean thickness and volume of total cerebellum, and cerebellar lobules) were compared in preSCA2 and SCA2 groups with CTR using Wilcoxon rank-sum test.

Differences with CTR were expressed as percentage, using the following formula: [(Median_Participant group_ – median_CTR_)/median_CTR_]·100, where the participant group was preSCA2 or SCA2.

To detect changes between baseline and 1-year follow-up evaluations within each subject category (i.e., preSCA2, SCA2, CTR), the Wilcoxon signed-rank test was used. The difference with baseline, was calculated as percentage of change using the following formula: [(Median _at1−year follow−up_ – median _at baseline_)/median _at baseline_]·100.

To explore the correlation between baseline measures and disease duration, we calculated Spearman's rank correlation coefficient. Correlations were calculated separately for the group of SCA2 patients and for preSCA2 subjects. For patients we used disease duration calculated as the difference between age at enrollment and age of ataxia manifestation. For pre-manifest subjects, we considered years-to-disease onset, calculated on the basis of CAG repeat length (26). We also analyzed the evolution of clinical and sMRI outcome measures combining preSCA2 and SCA2 groups and considering, as a unified variable of time, the years of disease duration for the patients and years before onset in premanifest SCA2 gene carriers. As exploratory analyses, we tested for linear, quadratic and cubic effects of time and chose the best fitting model.

The standardized response means (SRM), calculated as mean score change/standard deviation of change, was indicated as effect size index to enable comparison between scale and volumetric measures (32).

*P*-values of <0.05 were considered as statistically significant. Statistical correction for multiple comparisons was not applied owing to the small sample size and the exploratory nature of the study. Statistical analyses were conducted using either SAS software, version 9.4 or JMP® version 11.0.

## Results

### Baseline Evaluations

Among the 44 screened subjects, 42 were enrolled for the study. One screened SCA2 patient was not enrolled because refused MRI due to claustrophobia, and one preSCA2 subject was excluded because neuroimaging investigations showed the presence of Arnold-Chiari malformation. Baseline clinical characteristics of participants are summarized in Table 1A. At enrollment SCA2 patients had a mean age of 39 years, and a disease duration of 6.8 years. PreSCA2 subjects had a mean age of 39 years, and they were very far from disease onset with a median estimated time before symptoms of 16.6 years (range 10–18). The CTR group had a men age of 33 years.

**Table 1A:** Baseline demographic and clinical characteristics of participants.

**Clinical-genetic data**	**Controls (*n* = 15)**	**preSCA2 (*n* = 13)**	**SCA2 (*n* = 14)**
Gender (Female/Male)	12/3	9/4	4/10
Age (years)	32.8 (26.6–38.6)	39.9 (28.9–44.4)	38.8 (32.9–45.8)
CAG repeats on longer allele	–	36 (35–38)	40 (37–43)
Disease duration (years)	–	–	6.8 (2.7–10.8)
Estimated years-to-onset	–	15.5 (18.4–10.3)	
**Clinical and cognitive scores**			
SARA score	0.0 (0.0–0.0)	0.0 (0.0–1.0)	9.3 (4.5–14.0)**
INAS (Total count)	0 (0–0)	0 (0–1.0)^*^	2.0 (1.0–4.3)^**^
MMSE	30 (27.6–30.0)	30 (27.0–30.0)	27.6 (26.6–28.0)^*^
Digit span forward	5.9 (5.5–6.6)	5.7 (5.4–5.9)	5.9 (5.7–6.7)
SDMT	59.0 (50.0–65.0)	48 (42.0–52.0)^*^	39.5 (28.0–43.0)^**^
Phonemic fluency	33.0 (29.0–41.0)	34.0 (27.0–39.0)	26.0 (22.0–38.0)
Semantic fluency	47.3 (34.7–53.0)	39.3 (35.5–48.6)	35.4 (34.3–45.2)
ROCF-copy	36.0 (36.0–36.0)	36.0 (33.0–36.0)	36.0 (33.8–36.0)
ROCF-delayed recall	19.3 (15.3–22.8)	17.0 (13.8–17.8)	15.6 (9.8–23.0)

Variables are shown as median and interquantile range in parenthesis (Q1–Q3). SARA, Scale for Assessment and Rating of Ataxia; INAS, Inventory of Non-Ataxia Signs; SDMT, Symbol Digit Modalities Test; MMSE, Mini-Mental State Examination; ROCF, Rey-Osterrieth Complex Figure. The Wilcoxon rank-sum test for quantitative data, were used to detect differences between SCA2 groups and controls:

* p-value < 0.05 compared to controls;

** *p-value < 0.001 compared to controls*.

As expected, SARA score was higher in SCA2 (median 9.25 points) in comparison with CTR (*p* < 0.0001), and did not differ between preSCA2 and CTR. INAS total count was higher than CTR both in SCA2 (*p* < 0.0001) and preSCA2 subjects (*p* = 0.048). The most frequent non-ataxia signs observed in SCA2 patients were brainstem oculomotor abnormalities (86%), areflexia (43%), extensor plantar reflex (29%), and urinary dysfunction (29%). In preSCA2 only areflexia and extensor plantar reflex were reported (both 15%). In addition, muscle cramps were reported in 57% of patients, 15% of preSCA2, and 13% of CTR.

Cognitive tests indicated preserved intellectual levels in all participant groups. MMSE scores, though within normal reference values, were lower in SCA2 patients than in CTR (−8%, *p* = 0.026). Both SCA2 and preSCA2 had reduced SDMT scores in comparison with controls (−33%, *p* = 0.001 for SCA2, and 18.6%, *p* = 0.011 for preSCA2).

Cerebellar sMRI measures showed significantly reduced cortical cerebellar thickness (−13%) and reduced total cerebellar volume (−22%) in SCA2 patients in comparison with CTR (*p* < 0.0001 for both) (Table 1B). All cerebellar lobules (except lobule IX), showed significant volume loss in comparison with CTR. The lobules with the most severe degree of atrophy were lobules I–II (−35.7% mean volume compared with CTR), lobule IV (−29.4%), and lobule VI (−31.6%) (Supplementary Table 1A).

**Table 1B:** Baseline structural MRI characteristics of participants.

	**Participant groups^**°**^**	**Baseline**	Δ% with CTR^*^	**Group effect^**§**^**
		**Median (Q1–Q3)**		
**Cerebellum**				
Total cerebellar volume^#^	CTR	9.59 (8.80–9.92)		
	PreSCA2	8.94 (8.63–9.59)	−6.7	0.25
	SCA2	7.47 (6.88–7.85)	**−22.1**	**<0.0001**
Total cortical thickness^∞^	CTR	4.65 (4.54–4.72)		
	PreSCA2	4.59 (4.43–4.63)	−1.3	0.23
	SCA2	4.03 (3.83–4.13)	**−13.3**	**<0.0001**
**Subcortical brain structures**^#^				
Brain medulla	CTR	0.29 (0.27–0.34)		
	PreSCA2	0.28 (0.26–0.30)	−3.2	0.25
	SCA2	0.23 (0.22–0.24)	**−19.9**	**<0.0001**
Pons	CTR	0.93 (0.82–1.02)		
	PreSCA2	0.84 (0.77–0.89)	**−9.8**	**0.010**
	SCA2	0.47 (0.44–0.54)	**−49.2**	**<0.0001**
Superior cerebellar peduncles	CTR	0.015 (0.013–0.017)		
	PreSCA2	0.013 (0.012–0.015)	−8.3	0.17
	SCA2	0.008 (0.008–0.010)	**−42.3**	**<0.0001**
Midbrain	CTR	0.34 (0.32–0.39)		
	PreSCA2	0.35 (0.34–0.37)	4.8	0.59
	SCA2	0.34 (0.31–0.34)	0.05	0.33
Thalamus	CTR	0.48 (0.47–0.53)		
	PreSCA2	0.49 (0.48–0.54)	2.3	0.50
	SCA2	0.45 (0.41–0.46)	**−7.2**	**0.001**
Caudate	CTR	0.22 (0.21–0.24)		
	PreSCA2	0.21 (0.19–0.22)	−7.9	0.17
	SCA2	0.20 (0.19–0.22)	−11.0	0.06
Putamen	CTR	0.30 (0.29–0.31)		
	PreSCA2	0.30 (0.28–0.32)	0.9	0.56
	SCA2	0.27 (0.26–0.29)	**−9.8**	**0.006**
Pallidum	CTR	0.13 (0.12–0.14)		
	PreSCA2	0.12 (0.12–0.13)	−6.1	0.09
	SCA2	0.11 (0.10–0.12)	**−11.8**	**0.003**
Corpus callosum	CTR	0.27 (0.25–0.31)		
	PreSCA2	0.25 (0.24–0.29)	−5.4	0.24
	SCA2	0.22 (0.21–0.25)	−16.6	**0.007**

° Controls (CTR) n.15; Pre-symptomatic (preSCA2) n.13; affected patients (SCA2) n.14.

# Values are expressed as % of Total Intracranial Volume.

∞ Cortical thickness is expressed in millimeters (mm).

* For difference with controls the following formula was used: [(Median_Participant group (preSCA2 or SCA2)_−median_CTR_)/ median_CTR_]·100.

§ *Exact p-value: The Wilcoxon rank-sum test was used to detect participant groups effect respect to CTR. Values in bold represents, p < 0.05*.

Significant decrease in cerebellar cortical thickness was also found in all lobules, and in particular in lobules I-II (−22%), CrusII (−19%,), VIIIB (−27%), lobule IX (−32%), and lobule X (−57.8%) (Supplementary Table 1B). In SCA2 patients, sMRI measures of subcortical structures showed severe volume loss in the pons (−49% compared with CTR, *p* < 0.0001), superior cerebellar peduncles (SCP) (−42%, *p* < 0.0001), brain medulla (−20%, *p* < 0.0001), putamen and pallidum nuclei (−9.8%, *p* = 0.006; and −11.8%, *p* = 0.003), and corpus callosum (−16.6%, *p* = 0.007).

In preSCA2 subjects, cerebellar volume and cortical thickness did not differed from those of CTR. sMRI of subcortical brain structures showed a decrease in pons volume in comparison with CTR (−9.8%, *p* = 0.01) (Table 1B).

**Figure 1:**
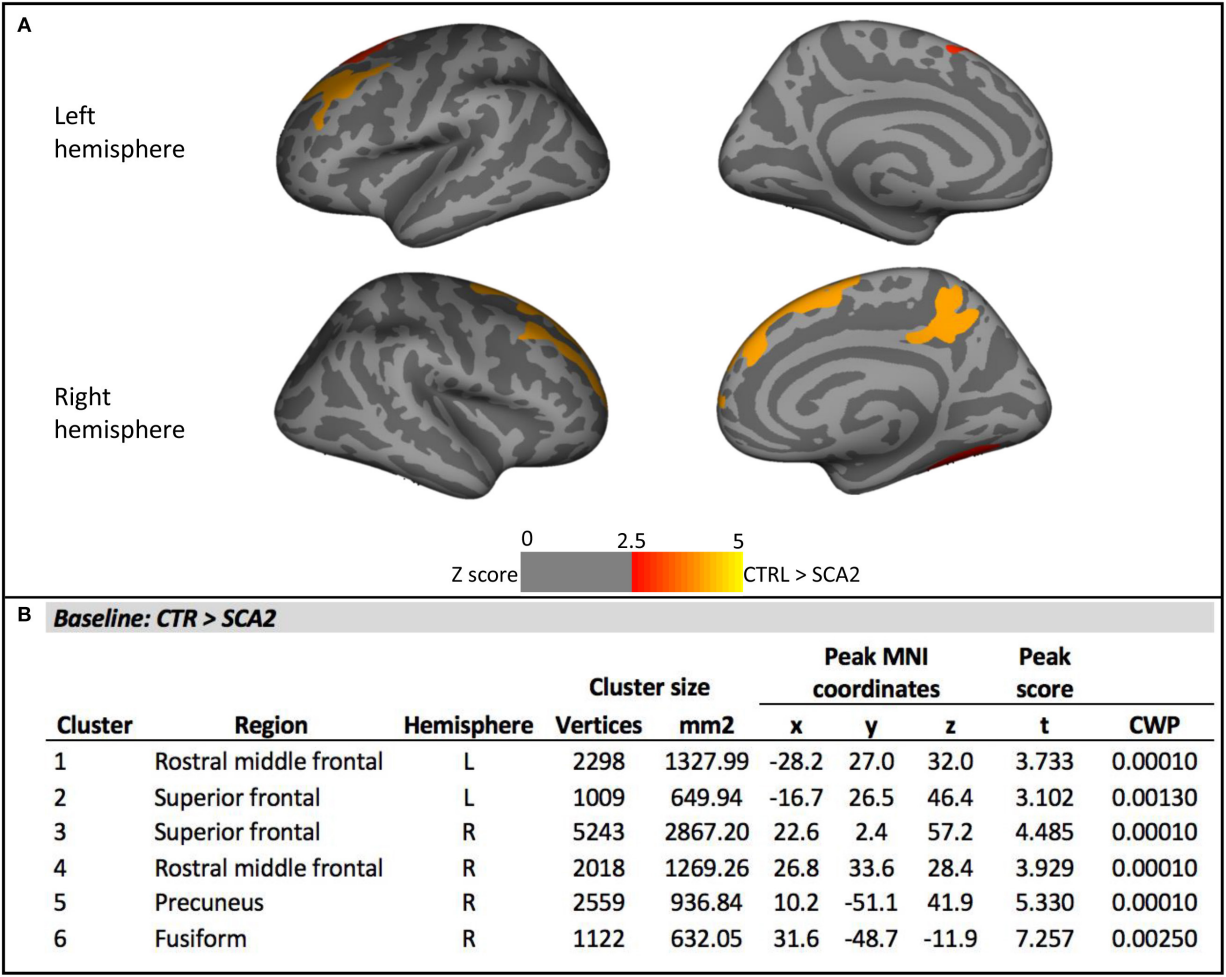
Vertex-wise analysis of cortical thickness at baseline. **(A)** Significant cortical thickness changes in the SCA2 patients compared to the healthy controls (CTR). All results were reported with *p* < 0.01, corrected for multiple comparisons using a Monte Carlo cluster-based simulation. Clusters color-coded in red indicate significantly decreased cortical thickness in the patient group compared to control (CTR). **(B)** Clusters showing significant cortical thickness changes in the SCA2 patients compared to the healthy CTR. CWP, *p*-value of the cluster (cluster-wise *p*-value); L, left; R, right. Peak coordinates are reported according to MNI305 space.

Vertex-wise analysis of cortical thickness showed significant differences between SCA2 patients and CTR in bilateral frontal regions comprising rostral middle frontal, caudal middle frontal, superior frontal areas, and in right precuneus and fusiform regions (Figure 1). Mean cortical thickness extracted from these cortical ROIs confirmed the significant differences between SCA2 and CTR, and a gradual reduction from preSCA2 to SCA2. In preSCA2 subjects, we observed a significant difference in bilateral rostral middle frontal regions (−6.5% thickness in the left side, and −4.5% in the right side) and in right precuneus (−6.9%) in comparison with CTR (Table 1C).

**Table 1C:** Baseline cortical clusters of vertex-analysis measures.

	**Participant groups^**°**^**	**Baseline**	Δ% with CTR^*^	**Group effect ^**§**^**
		**Median (Q1–Q3)**		
**Cortical cluster of vertex-analysis**^**∞**^				
Left rostral middle frontal	CTR	2.96 (2.89–3.06)		
	PreSCA2	2.77 (2.70–2.92)	**−6.5**	**0.030**
	SCA 2	2.70 (2.57–2.78)	−8.9	**<0.0001**
Left superior frontal	CTR	3.16 (3.06–3.31)		
	PreSCA2	3.02 (2.89–3.19)	−4.3	0.60
	SCA 2	2.80 (2.68–2.93)	−11.3	**<0.0001**
Right superior frontal	CTR	3.18 (3.12–3.32)		
	PreSCA2	3.06 (2.99–3.13)	−3.7	0.07
	SCA2	2.76 (2.66–2.89)	−13.3	**<0.0001**
Right rostral middle frontal	CTR	2.88 (2.80–2.92)		
	PreSCA2	2.75 (2.62–2.78)	**−4.5**	**0.018**
	SCA2	2.58 (2.41–2.58)	−10.4	**<0.0001**
Right precuneus	CTR	2.83 (2.76–2.90)		
	PreSCA2	2.64 (2.46–2.68)	**−6.9**	**0.021**
	SCA2	2.48 (2.36–2.58)	−12.6	**<0.0001**
Right fusiform	CTR	3.15 (3.11–3.22)		
	PreSCA2	3.01 (2.83–3.16)	−4.5	0.16
	SCA2	2.67 (2.46–2.80)	−15.4	**<0.0001**

° Controls (CTR) n.15; Pre-symptomatic (preSCA2) n.13; affected patients (SCA2) n.14.

* For difference with controls, the following formula was used: [(Median_Participant group_−median_CTR_)/ median_CTR_]·100, where the participant group was preSCA2 or SCA2.

∞ Cerebral cortical thickness is expressed in millimeters (mm).

§ *Exact p-value: The Wilcoxon rank-sum test was used to detect participant groups effect respect to CTR. In bold, p values < 0.05*.

### Longitudinal Evaluations

At 1-year follow up, SCA2 patients showed a mean increase of 1.0 point in SARA score, and a mean increase of 0.86 point in INAS count, but the differences with baseline evaluations were not significant.

Within-group longitudinal analyses of cognitive tests in SCA2 patients showed no effect of time, except for the ROCF-copy score that was significantly decreased at 1-year interval (*p* = 0.004). PreSCA2 subjects showed no changes in clinical and cognitive quantitative scores (Table 2).

**Table 2:** Clinical and MRI longitudinal changes at 1-year follow-up in patients and presymptomatic SCA2 subjects.

	**Presymptomatic SCA2 (n.9)**			**Patients SCA2 (n.14)**		
	**Median (Q1–Q3)**	% change with baseline^*^	***p*-Value time effect^**∞**^**	**Median (Q1–Q3)**	% change with baseline^*^	***p*-Value time effect^**∞**^**
**Clinical-cognitive scores**						
SARA	0.0 (0.0–0.5)	0	–	9.3 (5.0–16.0)	0	0.17
INAS (total count)	0.0 (0.0–2.0)	0	–	2.5 (1.0–5.0)	25.0	0.34
MMSE	28.6 (27.0–30.0)	5.1	1.0	27.9 (26.0–28.4)	1.1	0.88
Digit span forward	5.7 (5.5–7.1)	0.5	0.30	5.7 (5.6–5.8)	−4.2	0.34
SDMT	55.0 (49.0–59.0)	7.8	0.07	36.5 (25.0–43.0)	−7.6	0.23
Phonemic fluency	35.0 (27.5–42.5)	2.9	0.41	28.0 (23.0–39.0)	7.7	0.98
Semantic fluency	48.2 (29.5–51.7)	25.8	0.93	44.4 (28.1–49.6)	25.4	0.49
ROCF-copy	36.0 (34.6–36.0)	0	–	32.6 (30.7–36.0)	**−8.6**	**0.016**
ROCF-delayed recall	16.8 (9.6–20.9)	−1.5	0.89	14.1 (11.7–19.1)	−9.6	0.39
**Cerebellum**						
Total cerebellar volume^#^	9.04 (8.76–9.60)	−0.1	**0.004**	7.32 (6.79–7.74)	−2.1	**0.002**
Total cortical thickness^§^	4.68 (4.60–4.89)	1.7	0.027	4.07 (4.01–4.14)	1.1	0.81
**Subcortical brain structures**^**#**^						
Brain medulla	0.29 (0.28–0.30)	0.24	0.43	0.23 (0.22–0.24)	0.6	0.95
Pons	0.90 (0.83–0.92)	2.5	0.65	0.44 (0.40–0.52)	−5.5	**<0.0001**
SCP	0.014 (0.013–0.015)	−1.1	0.82	0.007 (0.007–0.009)	−14.6	**0.002**
Midbrain	0.37 (0.36–0.39)	6.0	0.16	0.30 (0.29–0.33)	−10.0	**0.001**
Thalamus	0.50 (0.50–0.51)	−1.7	0.50	0.43 (0.42–0.46)	−3.9	0.71
Caudate	0.20 (0.19–0.22)	−0.01	0.82	0.19 (0.19–0.22)	−2.3	0.71
Putamen	0.30 (0.27–0.31)	0.04	0.43	0.27 (0.25–0.28)	2.2	0.33
Pallidum	0.12 (0.11–0.13)	−0.6	0.43	0.12 (0.10–0.13)	4.0	0.86
Corpus callosum	0.25 (0.23–0.28)	1.9	0.43	0.23 (0.20–0.26)	0.5	0.30
**Brain cortical clusters thickness**^§^						
Left rostral middle frontal	2.82 (2.80–2.89)	−0.1	0.65	2.58 (2.55–2.76)	−4.5	0.74
Left superior frontal	3.15 (2.99–3.22)	−0.7	1.0	2.83 (2.62–2.87)	1.3	0.50
Right superior frontal	3.03 (3.02–3.12)	−1.1	0.16	2.82 (2.64–2.96)	2.4	0.31
Right rostral middle frontal	2.79 (2.57–2.81)	1.3	0.65	2.63 (2.43–2.72)	1.7	0.09
Right precuneus	2.65 (2.61–2.71)	0.6	0.91	2.52 (2.38–2.65)	1.6	0.46
Right fusiform	2.88 (2.82–3.11)	−4.4	0.36	2.83 (2.56–2.88)	6.0	0.22

# Values are expressed as % of Total Intracranial Volume.

§ Brain cortical clusters, obtained by vertex analysis: one SCA2 patient was excluded due to fail of white matter/gray matter segmentation in Freesurfer (values represent millimeters, mm).

* Difference with baseline, the following formula was used: [(Median_1−year follow−up_−median_baseline_)/median_baseline_]·100.

∞ *p-value: Wilcoxon signed-rank test was used to detect time effect, between baseline and follow-up examinations. In bold, p values < 0.05*.

Volumetric MRI longitudinal analyses showed no volume reduction in any of the analyzed areas for the healthy control group (Supplementary Table 4), and a progression of brain atrophy in SCA2 patients with significant time effect for cerebellar total volume (−2.1%, *p* = 0.002), and cerebellar lobule V (−6.5%,), lobule VI (−3.3%), and lobule VIIIA (−3%) (Supplementary Table 2A). Moreover, pons (−5.5%, *p* = 0.0001), SCP (−14.6%, *p* = 0.002), and midbrain (−10%, *p* < 0.001) showed significant changes between baseline and follow-up (Table 2).

In preSCA2, sMRI measures did not show significant differences except for total cerebellar volume, that was minimally but significantly reduced in comparison with baseline (*p* = 0.022).

In both groups, SCA2 and preSCA2, no longitudinal changes were observed for cerebellar cortical thickness (Supplementary Table 2B). For vertex longitudinal analyses, one SCA2 patient was excluded due to fail of white matter/gray matter segmentation in Freesurfer. Comparison between baseline and follow-up showed no significant changes over time in cerebral cortical thickness.

### Brain Imaging and Clinical Correlations

**Figure 2:**
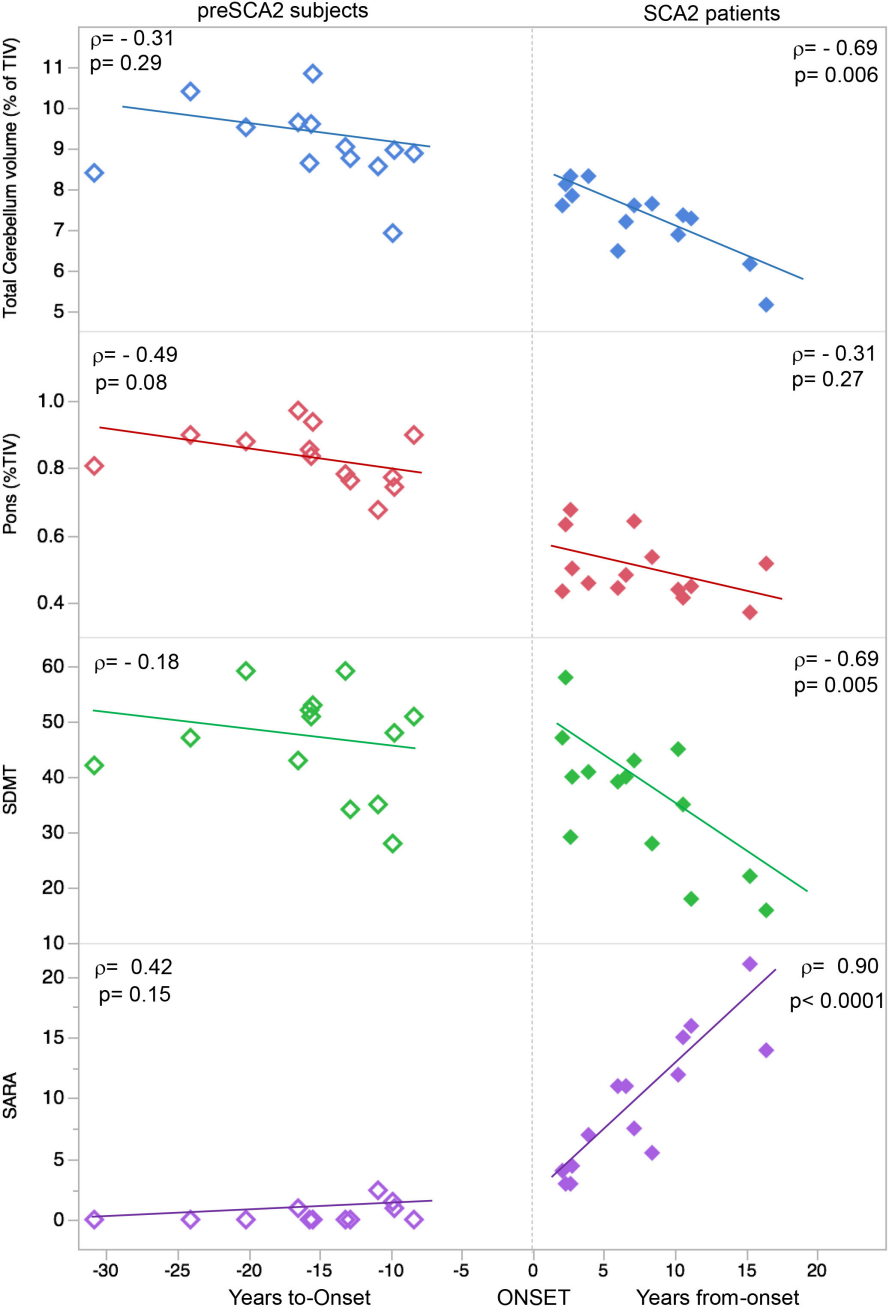
Correlations between clinical and sMRI measures. Graphical representation of the linear correlations between structural MRI-based pons and cerebellar volumes, Symbol Digit Modalities Test (SDMT), and Scale for Assessment and Rating of Ataxia (SARA) and time. In SCA2 patients, positive values indicate the year of disease duration after onset of ataxia. In presymptomatic SCA2 subjects, the negative values indicate the years before predicted age of onset. The rates of progression for clinical, cognitive, and sMRI measures are different in the different disease stages. Pons and Cerebellar volumes are indicated as percentage of total intracranial volume (TIV).

To investigate the rate of decline during the symptomatic and pre-manifest phases of the disease, we analyzed the correlations between disease duration and clinical and sMRI measures. We analyzed only the quantitative data that, at baseline, were significantly different in SCA2 patients in respect to CTR. In the group of SCA2 patients, years of disease duration correlate with SARA scores (ρ = 0.90; *p* < 0.0001), with SDMT scores (ρ = −0.69; *p* = 0.005), MMSE (ρ = −0.56, *p* = 0.036), total cerebellar volume (ρ = −0.69; *p* = 0.006), and mean cortical cerebellar thickness (ρ = −0.64; *p* = 0.014) (Figure 2). In addition, correlations with ρ > 0.3, could be detected for pons volume (ρ = −0.31; *p* = 0.27), brain medulla (ρ = −0.32; *p* = 0.26), and SCP (ρ = −0.38; *p* = 0.17).

The same measures considered in patients were tested in pre-manifest subjects. The estimated time-to-onset was used for the variable of time. In preSCA2, years-to-onset were significant correlated with cerebellar cortical thickness (ρ = −0.61; *p* = 0.027). Trends toward pathological changes in clinical scores (SARA and SDMT), and volume changes (pons and cerebellum) were observed in presymptomatic subjects with proximity to disease onset (Figure 2).

**Figure 3:**
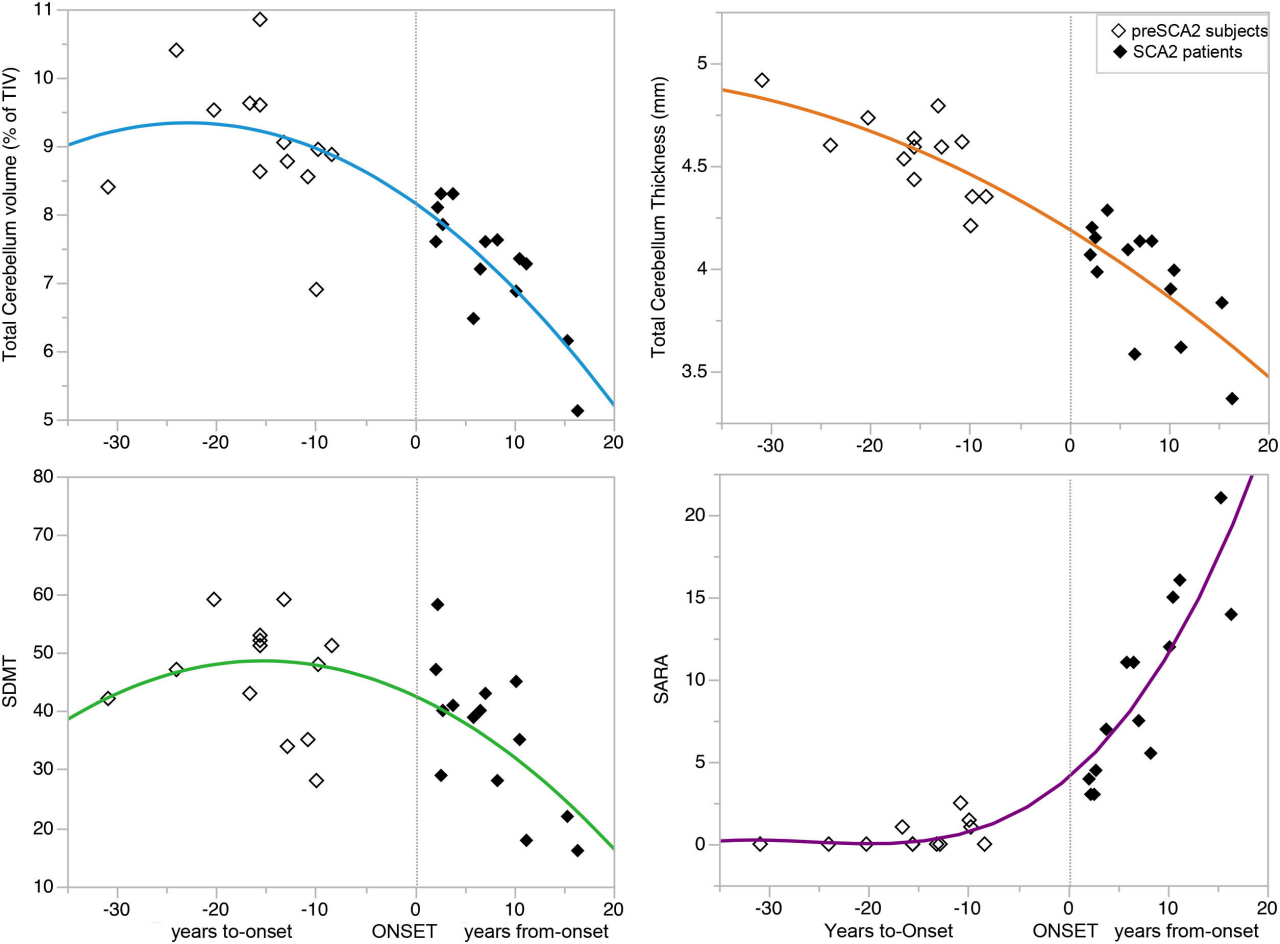
Progression of disease features during the preclinical and clinical disease phases. The curves showed the progression of clinical (SARA and SDMT scores) and MRI variables total cerebellar volume and cortical thickness) during the preclinical and clinical disease phases of the disease. The non-linear relationships showed no progression of the variables in the pre-manifest phase far from disease onset, minimal progression in the decade preceding the onset, and marked progression after symptoms manifestation. See also Supplementary Table 3 for the parameters of the models and *p*-values. Symbol Digit Modalities Test (SDMT), and Scale for Assessment and Rating of Ataxia (SARA).

Changes in outcome measures showed different rates of progression in the symptomatic and in the presymptomatic phases, with very low progression of cerebellar atrophy and ataxia clinical score in the preclinical phase, and with more rapid decline after the full manifestation of the disease (Figure 3) (Supplementary Table 3).

The evaluations of SRM for clinical and sMRI measures showed that changes of SARA and INAS scores over time had small effect size in both SCA2 patients (SRM: 0.39 and 0.33), and in preSCA2 subjects (SRM: 0.33) (Table 3). sMRI metrics demonstrate large effect sizes for total cerebellar volume in both preSCA2 (SRM: −0.97) and in affected SCA2 patients (SRM: −1.12), while brainstem volumes, and in particular pons volume, had a large effect size in patients (SRM = −2.16), but not in the preclinical subjects (SRM = 0.18).

**Table 3:** Standardized response means (SRM) estimated for selected clinical and neuroimaging measures.

	**preSCA2 SRM**	**SCA2 SRM**
SARA	0.33	0.39
INAS	0.23	0.33
SDMT	0.74	−1.00
Total cerebellar volume	−0.97	−1.12
Cerebellar cortical thickness	1.00	0.26
Brainstem volume	0.43	−1.93
Midbrain	0.56	−1.69
SCP	−0.10	−0.81
Pons	0.18	−2.16
Medulla	0.21	−0.05

*SARA, scale for the assessment and rating of ataxia; INAS, Inventory of Non-Ataxia Symptoms; SDMT, Symbol Digit Modalities Test. The cut-offs values for the evaluation of the effect size for the specified measures were set as: 0.2 = small; 0.5 = medium; 0.8 = large; 1.2 = very large effect size*.

## Discussion

In this study, we prospectively followed a cohort of preclinical and early symptomatic SCA2 mutation carriers, over 1-year period, using quantitative clinical and brain MRI measures. SARA score efficiently followed the progression of ataxia in SCA2 patients, with a mean increase of 1.0 point at 1-year longitudinal evaluation, but the scale was not effective in detecting abnormalities or progression in preSCA2 subjects. These figures are in agreement with previous observational studies showing a yearly increase of 1.0–1.75 point of the SARA scores in patients. The progression rates were shown to be different in the early and in the late stages of the disease (33, 34). In preclinical subjects, we confirmed a non-linear progression of SARA, with increasing scores around the time of disease onset (18, 35–39) (Figure 3).

The inventory of non-ataxia signs (i.e., INAS total count), differently than SARA, revealed a small but noticeable difference between preSCA individuals and healthy controls. In agreement with previous observations, we found that muscle cramps, areflexia, and extensor plantar reflex are present in preSCA2 subjects many years before the ataxic signs (4, 17, 22).

Cognitive evaluations showed that both SCA2 patients and preSCA subjects had significant lower scores than CTR in SDMT. SDMT has not been extensively used in SCAs, but is has been proposed as a cognitive outcome measures in several neurological disorders, including Huntington disease, Friedreich ataxia and Fragile X-Associated Tremor/Ataxia syndrome (40–42). SDMT is a measure of speed and efficiency in multiple cognitive processes involving memory, word retrieval, and executive function. We hypothesize that this test may be a sensitive measure to detect very early deficits of executive functions also in SCA patients, but further studies are needed to confirm this preliminary observation. In the battery of cognitive tests, a time-effect in the was identified only for the ROCF-copy score for SCA2 patients. Previous longitudinal studies in symptomatic patients have also shown very slow progression of cognitive decline (24, 43).

In preclinical SCA2 mutation carriers, cognitive characteristics have been extensively studied only in a large cohort from Cuba (4). This latter study showed executive function impairment in preSCA2, however, due to different inclusion criteria, the enrolled cohort included a percentage of subjects with overt ataxic signs (SARA up to 4 points).

The neuroimaging data of our study largely confirmed previous finding showing that cerebellum, brain medulla, pons, and superior cerebellar peduncles are consistently affected in SCA2 patients, giving the characteristic imaging of olivo-ponto-cerebellar atrophy (OPCA) (10–13, 16, 44–48). We also confirmed that atrophy was more severe in cerebellar lobules of the anterior lobe and superior posterior lobe, predominantly associated with motor control (17). Our study also confirmed, in symptomatic patients, volume loss in basal ganglia (thalamus, putamen, and pallidum) and corpus callosum (12, 13, 47). At 12-months-longitudinal evaluations, we observed a significant increment of the atrophy both in cerebellar and pontine regions, and in subcortical structures. Similar findings were previously reported in longitudinal studies having much longer follow-up intervals (range 2–6 years) (49, 50).

Only few MRI reports are available for the presymptomatic phase (4, 16, 17). These observational studies revealed mild cerebellar and brainstem volume loss in subjects carrying the disease mutation, with volume loss being more pronounced in the individuals closest to the expected onset of ataxia (16). One longitudinal MRI study, performed in a subgroup of 5 SCA2 gene carriers, showed significant decrease in cerebellar and pons volumes at 6-years interval (18). In our cohort of preSCA2 subjects, we found that the pons was the brain structure primarily affected by volume decrease in respect to healthy CTR, but at 12-months interval no significant progression of the atrophy could be detected. On the other hand, total cerebellum volume did not differ from healthy controls, both at baseline and at follow-up, but within the preSCA2 group, we observed a mild, but significant, decrease in total cerebellar volume at longitudinal analysis.

On the contrary, analyses of cerebellar cortical thickness failed to show significant effect of time within SCA2 groups. This is a major novelty of our study, since there are no previous data reporting changes in cerebellar cortical thickness for pre-manifest or SCA2 patients. Our findings are analogous to the data reported in SCA1 and SCA3 diseases, in which cerebellar cortical thinning was detected only in affected patients with a disease duration of at least 5 years (51, 52). Our SCA2 patients had a mean disease duration of 6.8 years, and they present significant reduction of cortical cerebellar thickness both at baseline and follow-up measures in comparison with controls, but no measurable change could be detected at 12-months evaluation. These data suggest that: (1) cerebellar cortex is part of the neurodegenerative pattern in SCA2, analogously to SCA1 and SCA3 diseases, (2) cortical thinning is detectable only several years after disease onset, and (3) the progression of cerebellar cortex thinning appears much slower than that observed for cerebellar volumes. Overall these data suggest that cerebellar volume rather than thickness would be a better biomarker to track the progression of SCA2 disease, including the pre-symptomatic phase.

It has to be noted that in our study all preSCA2 subjects were very far from disease onset (median 16 years, range 8–30 years). This aspect was not predetermined by our inclusion-exclusion criteria, but was unintentional due to unbiased recruitment of the at-risk relatives belonging to our SCA2 families. The advantage of this cohort was to have the opportunity of studying a very large time-window of the pre-ataxic stage, the disadvantage was the large heterogeneity.

We also have to consider the limitations of this work, mainly represented by the small sample size and the high rate of dropout in preclinical SCA2 participants at longitudinal evaluation. Both factors considerably reduce the study power, and may affect statistical analyses particularly in the preSCA population. Though these limitations highlight the need of multicenter studies, our results support the importance of specific clinical and MRI longitudinal observations in SCA gene carriers. Particularly, in preSCA2 cerebellar and pons volume loss is detectable ~10-years from expected disease manifestation. Our extensive analysis across the whole brain, allowed us to demonstrate a significant trend of cortical thinning in bilateral rostral-middle frontal and in right precuneus in preSCA2 subjects, suggesting that also these cortical areas could also be affected very early in the course of the disease. The rate of progression of cerebellar volume reduction is very different in the period before and after clinical onset, but it can be appreciated at 1-year interval. Structural MRI of these specific areas may allow early quantification of neurodegeneration, and may represent possible outcome measures to implement trial readiness for upcoming disease-modifying clinical trials.

## Data Availability Statement

The raw data supporting the conclusions of this article will be made available by the authors, without undue reservation.

## Ethics Statement

The studies involving human participants were reviewed and approved by Comitato Etico Regione Lombardia Sezione Fondazione IRCCS Istituto Neurologico “Carlo Besta”. The patients/participants provided their written informed consent to participate in this study.

## Author Contributions

AN, LS, and CM: conception, execution, writing of the draft, and review manuscript. AM, AC, LN, CP, SF, MG, MB, CG, and FT: conception, execution, and writing of the draft. LP: statistical analyses, writing of the draft, and review manuscript. All authors contributed to the article and approved the submitted version.

## Conflict of Interest

The authors declare that the research was conducted in the absence of any commercial or financial relationships that could be construed as a potential conflict of interest.

## References

[1] Klockgether T Mariotti C Paulson HL Spinocerebellar ataxia. Nat Rev Dis Primers. (2019) 5:24 10.1038/s41572-019-0074-3 30975995

[2] Ruano L Melo C Silva MC Coutinho P. The global epidemiology of hereditary ataxia and spastic paraplegia: a systematic review of prevalence studies. Neuroepidemiology. (2014) 42:174–83. 10.1159/000358801 24603320

[3] Furtado S Payami H Lockhart PJ Hanson M Nutt JG Singleton AA. Profile of families with parkinsonism-predominant spinocerebellar ataxia type 2 (SCA2). Mov Disord. (2004) 19:622–9. 10.1002/mds.20074 15197699

[4] Velázquez-Pérez L Rodríguez-Labrada R Cruz-Rivas EM Fernández-Ruiz J Vaca-Palomares I Lilia-Campins J. Comprehensive study of early features in spinocerebellar ataxia 2: delineating the prodromal stage of the disease. Cerebellum. (2014) 13:568–79. 10.1007/s12311-014-0574-3 24906824

[5] Velázquez-Pérez L Rodríguez-Labrada R Canales-Ochoa N Montero JM Sánchez-Cruz G Aguilera-Rodríguez R. Progression of early features of spinocerebellar ataxia type 2 in individuals at risk: a longitudinal study. Lancet Neurol. (2014) 13:482–9. 10.1016/S1474-4422(14)70027-4 24657153

[6] Velázquez-Pérez L Rodríguez-Labrada R Laffita-Mesa JM. Prodromal spinocerebellar ataxia type 2: prospects for early interventions and ethical challenges. Mov Disord. (2017) 32:708–18. 10.1002/mds.26969 28256108

[7] Maas RP van Gaalen J Klockgether T van de Warrenburg BP. The preclinical stage of spinocerebellar ataxias. Neurology. (2015) 85:96–103. 10.1212/WNL.0000000000001711 26062625

[8] Baldarçara L Currie S Hadjivassiliou M Hoggard N Jack A Jackowski AP. Consensus paper: radiological biomarkers of cerebellar diseases. Cerebellum. (2015) 14:175–96. 10.1007/s12311-014-0610-3 25382714 PMC4929983

[9] Mascalchi M Vella A. Neuroimaging biomarkers in SCA2 gene carriers. Int J Mol Sci. (2020) 21:1020. 10.3390/ijms21031020 32033120 PMC7037189

[10] Goel G Pal PK Ravishankar S Venkatasubramanian G Jayakumar PN Krishna N. Gray matter volume deficits in spinocerebellar ataxia: an optimized voxel based morphometric study. Parkinsonism Relat Disord. (2011) 17:521–7. 10.1016/j.parkreldis.2011.04.008 21600833

[11] Mercadillo RE Galvez V Díaz R Hernández-Castillo CR Campos-Romo A Boll MC. Parahippocampal gray matter alterations in spinocerebellar ataxia type 2 identified by voxel based morphometry. J Neurol Sci. (2014) 347:50–8. 10.1016/j.jns.2014.09.018 25263602

[12] Della Nave R Ginestroni A Tessa C Cosottini M Giannelli M Salvatore E. Brain structural damage in spinocerebellar ataxia type 2. A voxel-based morphometry study. Mov Disord. (2008) 23:899–903. 10.1002/mds.21982 18311829

[13] D'Agata F Caroppo P Boghi A Coriasco M Caglio M Baudino B. Linking coordinative and executive dysfunctions to atrophy in spinocerebellar ataxia 2 patients. Brain Struct Funct. (2011) 216:275–88. 10.1007/s00429-011-0310-4 21461742

[14] Salvatore E Tedeschi E Mollica C Vicidomini C Varrone A Coda AR. Supratentorial and infratentorial damage in spinocerebellar ataxia 2: a diffusion-weighted MRI study. Mov Disord. (2014) 29:780–6. 10.1002/mds.25757 24375449

[15] Inagaki A Iida A Matsubara M Inagaki H. Positron emission tomography and magnetic resonance imaging in spinocerebellar ataxia type 2: a study of symptomatic and asymptomatic individuals. Eur J Neurol. (2005) 12:725–8. 10.1111/j.1468-1331.2005.01011.x 16128876

[16] Reetz K Rodríguez-Labrada R Dogan I Mirzazade S Romanzetti S Schulz JB. Brain atrophy measures in preclinical and manifest spinocerebellar ataxia type 2. Ann Clin Transl Neurol. (2018) 5:128–37. 10.1002/acn3.504 29468174 PMC5817824

[17] Jacobi H Reetz K du Montcel ST Bauer P Mariotti C Nanetti L. Biological and clinical characteristics of individuals at risk for spinocerebellar ataxia types 1, 2, 3, and 6 in the longitudinal RISCA study: analysis of baseline data. Lancet Neurol. (2013) 12:650–8. 10.1016/S1474-4422(13)70104-2 23707147

[18] Jacobi H du Montcel ST Romanzetti S Harmuth F Mariotti C Nanetti L. Conversion of individuals at risk for spinocerebellar ataxia types 1, 2, 3, and 6 to manifest ataxia (RISCA): a longitudinal cohort study. Lancet Neurol. (2020) 19:738–47. 10.1016/S1474-4422(20)30235-0 32822634

[19] Scoles DR Meera P Schneider MD Paul S Dansithong W Karla P. Antisense oligonucleotide therapy for spinocerebellar ataxia type 2. Nature. (2017) 544:362–6. 10.1038/nature22044 28405024 PMC6625650

[20] Sequeiros J Seneca S Martindale J. Consensus and controversies in best practices for molecular genetic testing of spinocerebellar ataxias. Eur J Hum Genet. (2010) 18:1188–95. 10.1038/ejhg.2010.10 20179748 PMC2987480

[21] Schmitz-Hübsch T du Montcel ST Baliko L Berciano J Boesch S Depondt C. Scale for the assessment and rating of ataxia: development of a new clinical scale. Neurology. (2006) 66:1717–20. 10.1212/01.wnl.0000219042.60538.92 16769946

[22] Jacobi H Rakowicz M Rola R Fancellu R Mariotti C Charles P. Inventory of non-ataxia signs (INAS): Validation of a new clinical assessment instrument. Cerebellum. (2013) 12:418–28. 10.1007/s12311-012-0421-3 23090211

[23] Sheridan LK Fitzgerald HE Adams KM Nigg JT Martel MM Puttler LI. Normative symbol digit modalities test performance in a community-based sample. Arch Clin Neuropsychol. (2006) 21:23–8. 10.1016/j.acn.2005.07.003 16139470

[24] Fancellu R Paridi D Tomasello C Panzeri M Castaldo A Genitrini S. Longitudinal study of cognitive and psychiatric functions in spinocerebellar ataxia types 1 and 2. J Neurol. (2013) 260:3134–43. 10.1007/s00415-013-7138-1 24122064

[25] Olivito G Lupo M Iacobacci C Clausi S Romano S Masciullo M. Microstructural MRI basis of the cognitive functions in patients with spinocerebellar ataxia type 2. Neuroscience. (2017) 366:44–53. 10.1016/j.neuroscience.2017.10.007 29031602

[26] Tezenas du Montcel S Durr A Rakowicz M Nanetti L Charles P Sulek A. Prediction of the age at onset in spinocerebellar ataxia type 1, 2, 3 and 6. J Med Genet. (2014) 51:479–86. 10.1136/jmedgenet-2013-102200 24780882 PMC4078703

[27] Romero JE Coupé P Giraud R Ta VT Fonov V Park MTM. CERES: A new cerebellum lobule segmentation method. Neuroimage. (2017) 147:916–24. 10.1016/j.neuroimage.2016.11.003 27833012

[28] Iglesias JE Van Leemput K Bhatt P Casillas C Dutt S Schuff N. Bayesian segmentation of brainstem structures in MRI. Neuroimage. (2015) 113:184–95. 10.1016/j.neuroimage.2015.02.065 25776214 PMC4434226

[29] Iglesias JE Van Leemput K Augustinack J Insausti R Fischl B Reuter M. Bayesian longitudinal segmentation of hippocampal substructures in brain MRI using subject-specific atlases. Neuroimage. (2016) 141:542–55. 10.1016/j.neuroimage.2016.07.020 27426838 PMC5026967

[30] Fischl B Salat DH Busa E Albert M Dieterich M Haselgrove C. Whole brain segmentation: automated labeling of neuroanatomical structures in the human brain. Neuron. (2002) 33:341–55. 10.1016/s0896-6273(02)00569-x 11832223

[31] Buckner RL Head D Parker J Fotenos AF Marcus D Morris JC. A unified approach for morphometric and functional data analysis in young, old, and demented adults using automated atlas-based head size normalization: reliability and validation against manual measurement of total intracranial volume. Neuroimage. (2004) 23:724–38. 10.1016/j.neuroimage.2004.06.018 15488422

[32] Reetz K Costa AS Mirzazade S Lehmann A Juzek A Rakowicz M. Genotype-specific patterns of atrophy progression are more sensitive than clinical decline in SCA1, SCA3 and SCA6. Brain. (2013) 136:905–17. 10.1093/brain/aws369 23423669

[33] Diallo A Jacobi H Tezenas du Montcel S Klockgether T. Natural history of most common spinocerebellar ataxia: a systematic review and meta-analysis. J Neurol. (2020). 10.1007/s00415-020-09815-2. [Epub ahead of print]. 32266540

[34] Monte TL Reckziegel EDR Augustin MC Locks-Coelho LD Santos ASP Furtado GV. The progression rate of spinocerebellar ataxia type 2 changes with stage of disease. Orphanet J Rare Dis. (2018) 13:20. 10.1186/s13023-017-0725-y 29370806 PMC5785809

[35] Jacobi H Bauer P Giunti P Labrum R Sweeney MG Charles P. The natural history of spinocerebellar ataxia type 1, 2, 3, and 6: a 2-year follow-up study. Neurology. (2011) 77:1035–41. 10.1212/WNL.0b013e31822e7ca0 21832228 PMC3174068

[36] Jacobi H du Montcel ST Bauer P Giunti P Cook A Labrum R. Long-term disease progression in spinocerebellar ataxia types 1, 2, 3, and 6: a longitudinal cohort study. Lancet Neurol. (2015) 14:1101–8. 10.1016/S1474-4422(15)00202-1 26377379

[37] Lee YC Liao YC Wang PS Lee IH Lin KP Soong BW. Comparison of cerebellar ataxias: a three-year prospective longitudinal assessment. Mov Disord. (2011) 26:2081–7. 10.1002/mds.23809 21626567

[38] Tezenas du Montcel S Charles P Goizet C Marelli C Ribai P Vincitorio C. Factors influencing disease progression in autosomal dominant cerebellar ataxia and spastic paraplegia. Arch Neurol. (2012) 69:500–8. 10.1001/archneurol.2011.2713 22491195

[39] Ashizawa T Figueroa KP Perlman SL Gomez CM Wilmot GR Schmahmann JD. Clinical characteristics of patients with spinocerebellar ataxias 1, 2, 3 and 6 in the US; a prospective observational study. Orphanet J Rare Dis. (2013) 8:177. 10.1186/1750-1172-8-177 24225362 PMC3843578

[40] Tabrizi SJ Scahill RI Owen G Durr A Leavitt BR Roos RA. Predictors of phenotypic progression and disease onset in premanifest and early-stage Huntington's disease in the TRACK-HD study: analysis of 36-month observational data. Lancet Neurol. (2013) 12:637–49. 10.1016/S1474-4422(13)70088-7 23664844

[41] Saccà F Costabile T Abate F Liguori A Paciello F Pane C. Normalization of timed neuropsychological tests with the PATA rate and nine-hole pegboard tests. J Neuropsychol. (2018) 12:471–83. 10.1111/jnp.12125 28477351

[42] Hocking DR Loesch DZ Trost N Bui MQ Hammersley E Francis D. Total and regional white matter lesions are correlated with motor and cognitive impairments in carriers of the FMR1 premutation. Front Neurol. (2019) 10:832. 10.3389/fneur.2019.00832 31456732 PMC6700239

[43] Le Pira F Giuffrida S Maci T Marturano L Tarantello R Zappalà G. Dissociation between motor and cognitive impairments in SCA2: evidence from a follow-up study. J Neurol. (2007) 254:1455–6. 10.1007/s00415-007-0548-1 17680296

[44] Brenneis C Bösch SM Schocke M Wenning GK Poewe W. Atrophy pattern in SCA2 determined by voxel-based morphometry. Neuroreport. (2003) 14:1799–802. 10.1097/00001756-200310060-00008 14534423

[45] Guerrini L Lolli F Ginestroni A Belli G Della Nave R Tessa C Brainstem neurodegeneration correlates with clinical dysfunction in SCA1 but not in SCA2. A quantitative volumetric, diffusion and proton spectroscopy MR study. Brain. (2004) 127:1785–95. 10.1093/brain/awh201 15240431

[46] Jung BC Choi SI Du AX Cuzzocreo JL Ying HS Landman BA. MRI shows a region-specific pattern of atrophy in spinocerebellar ataxia type 2. Cerebellum. (2012) 11:272–9. 10.1007/s12311-011-0308-8 21850525 PMC3785794

[47] Mascalchi M Diciotti S Giannelli M Ginestroni A Soricelli A Nicolai E. Progression of brain atrophy in spinocerebellar ataxia type 2: a longitudinal tensor-based morphometry study. PLoS ONE. (2014) 9:e89410. 10.1371/journal.pone.0089410 24586758 PMC3934889

[48] Hernandez-Castillo CR Galvez V Mercadillo R Diaz R Campos-Romo A Fernandez-Ruiz J Extensive white matter alterations and its correlations with ataxia severity in SCA2 patients. PLoS ONE. (2015) 10:e0135449 10.1371/journal.pone.0135449 26263162 PMC4532454

[49] Mascalchi M Toschi N Giannelli M Ginestroni A Della Nave R Nicolai E. Progression of microstructural damage in spinocerebellar ataxia type 2: a longitudinal DTI study. Am J Neuroradiol. (2015) 36:1096–101. 10.3174/ajnr.A4343 25882284 PMC8013027

[50] Adanyeguh IM Perlbarg V Henry PG Rinaldi D Petit E Valabregue R. Autosomal dominant cerebellar ataxias: imaging biomarkers with high effect sizes. Neuroimage Clin. (2018) 19:858–67. 10.1016/j.nicl.2018.06.011 29922574 PMC6005808

[51] Martins Junior CR Martinez ARM Vasconcelos IF de Rezende TJR Casseb RF Pedroso JL. Structural signature in SCA1: clinical correlates, determinants and natural history. J Neurol. (2018) 265:2949–59. 10.1007/s00415-018-9087-1 30324307

[52] Rezende TJR de Paiva JLR Martinez ARM Lopes-Cendes I Pedroso JL Barsottini OGP. Structural signature of SCA3: from presymptomatic to late disease stages. Ann Neurol. (2018) 84:401–8. 10.1002/ana.25297 30014526

