# Supplementary material for: Progression of Cerebellar Atrophy in Spinocerebellar Ataxia Type 2 Gene Carriers: A Longitudinal MRI Study in Preclinical and Early Disease Stages
Source: Front Neurol. 2020 Dec 15;11:616419. doi: 10.3389/fneur.2020.616419 (PMC7770103; doi:10.3389/fneur.2020.616419)
Supplement: Supplementary file 1 [file Data_Sheet_1.docx]

**Progression of cerebellar atrophy in spinocerebellar ataxia type 2 gene carriers:**

**A longitudinal MRI study in preclinical and early disease stages**

Nigri A et al.

**Supplementary Material**

**MRI Methods**

***Freesurfer***

Cerebral cortex and subcortical nuclei were segmented using FreeSurfer software (Massachusetts General Hospital, Harvard Medical School; http://freesurfer.net, version 6) with the "recon–all" pipeline. Brainstem substructures (i.e. medulla oblongata, pons, midbrain, and superior cerebellar peduncles - SCP) were obtained adding the flag -brainstem-structures to your “recon-all” pipeline (Fischl et al. 2000).

For surface analysis, the pipeline included affine registration to the MNI305 atlas, a correction for B1 bias field, skull stripping, segmentation of subcortical white matter. Using cutting planes, right and left hemispheres were separated and cerebellum and brainstem removed. For each hemisphere a white matter surface was estimated based on MR image intensity gradients between the white and grey matter as well as a pial surface based on intensity gradients between the grey matter and cerebrospinal fluid (CSF). Expert operators performed visual inspection of white matter segmentations and pial boundary surfaces in order to detect major topological inaccuracies that were manually corrected by an expert operator (C.P.) blind to the individual category (i.e. CTR, pre-SCA2, SCA2). Cortical thickness can be calculated as the distance between the vertices of white and pial surface. “-qcache” flag was used for process and resample the individual cortical surfaces onto common (fsaverage) space characterized by 163,842 vertices per hemisphere to establish inter-individual correspondence.

For volume analysis, the pipeline included affine registration to the MNI305 (Collins et al 1994) atlas, a correction for B1 bias field, skull stripping, and segmentation of grey matter, white matter, CSF, and subcortical structure. The classification for each voxel was obtained based on prior probabilities. Subsequently, the subcortical segmentations (i.e. bilateral putamen, caudate, pallidum, thalamus, corpus callosum) were computed according to Fischl and colleagues (Fischl et al., 2002), while brainstem (i.e. medulla oblongata, pons, midbrain, and superior cerebellar peduncles) according to Iglesias and colleagues 2015 (Iglesias et al., 2015). All segmentations were visually checked for errors. No manual interventions were performed on the data. The volume of each segmented subcortical and brainstem regions and the estimated total intracranial volumes (TIV) were obtained (‘aseg’ file) (Buckner et al., 2004). Total volume of the corpus callosum was computed by summing the volumes of genu, body and splenium segments.

Finally, to estimate the differences in the cortical thickness at each vertex between SCA2 and CTR at baseline, a General Linear Model in a vertex-wise analysis was performed with “qdec” module of Freesurfer. Monte Carlo simulation as implemented in Freesurfer with a cluster- forming threshold of p<0.01 (two-sided) was used to correct for multiple comparisons. Significant clusters in this contrast map were neuroanatomically identified based on the Desikan-Killiany cortical atlas (Desikan et al., 2006) and identified as cortical ROIs. Mean cortical thickness for each cluster showing a significant difference between the SCA2 patients and CTR (i.e. cortical ROI) was extracted for each subject (CTR, preSCA2, and SCA2) at baseline and follow-up evaluation.

**Ceres**

A quantitative evaluation of cerebellum lobules was obtained using CERES automated cerebellum parcellation algorithm (Romero et al., 2017). With this tool, the 3D T1-weighted structural images are denoised, corrected for inhomogeneity, rigid-body registered to Montreal Neurological Institute (MNI) template, cropped around the cerebellum area, and normalized to the MNI cropped cerebellum atlas. Finally, An automatic multi-atlas patch-based segmentation was then applied to obtain cerebellar cortical thickness and volume (i.e. sum of grey and white matter), expressed in percentage of total intracranial volume, for each lobule (Manjón and Coupe, 2016).

**References**

Fischl B, Dale AM. Measuring the thickness of the human cerebral cortex from magnetic resonance images. Proceedings of the National Academy of Sciences. 2000. pp. 11050–11055. doi:10.1073/pnas.200033797

Iglesias JE, Van Leemput K, Bhatt P, et al. Bayesian segmentation of brainstem structures in MRI. NeuroImage 2015;113:184–195.

Iglesias JE, Van Leemput K, Augustinack J, et al. Bayesian longitudinal segmentation of hippocampal substructures in brain MRI using subject-specific atlases. NeuroImage 2016;141:542–555.

Fischl B, Salat DH, Busa E, et al. Whole Brain Segmentation: Automated Labeling of Neuroanatomical Structures in the Human Brain. Neuron 2002;33:341–355.

Buckner RL, Head D, Parker J, et al. A unified approach for morphometric and functional data analysis in young, old, and demented adults using automated atlas-based head size normalization: reliability and validation against manual measurement of total intracranial volume. NeuroImage 2004;23:724–738.

Desikan RS, Ségonne F, Fischl B, Quinn BT, Dickerson BC, Blacker D, Buckner RL, Dale AM, Maguire RP, Hyman BT, Albert MS, Killiany RJ. An automated labeling system for subdividing the human cerebral cortex on MRI scans into gyral based regions of interest. Neuroimage. 2006 Jul 1;31(3):968-80. doi: 10.1016/j.neuroimage.2006.01.021.

Romero JE, Coupé P, Giraud R, et al. CERES: A new cerebellum lobule segmentation method. NeuroImage 2017;147:916–924.

Manjón JV, Coupé P. volBrain: An Online MRI Brain Volumetry System. Front Neuroinform. 2016;10:30. Published 2016 Jul 27. doi:10.3389/fninf.2016.00030

**Supplementary Table 1a. Baseline sMRI cerebellar lobule volume in SCA2 gene mutation carriers compared with controls.**

|  | |  | **SCA2 presymptomatic subjects (n.13)** | | |  |  | **SCA2 Patients (n.14)** | | |  |
| --- | --- | --- | --- | --- | --- | --- | --- | --- | --- | --- | --- |
| **Cerebellar lobule**  **Volume^#^** | | **Median**  **(Q1-Q3)** | | ** **  **with**  **CTR^*^** | **p value^°°^** | | **Median**  **(Q1-Q3)** | | ****  **With CTR^*^** | **p value^°°^** | |
| **Anterior** | **I-II** | 0.009 (0.007-0.01) | | 2.3 | 0.69 | | 0.005 (0.004-0.007) | | **-35.7** | **0.002** | |
|  | **III** | 0.095 (0.09-0.11) | | 0 | ~~-~~ | | 0.073 (0.06-0.09) | | **-23.1** | **0.014** | |
|  | **IV** | 0.31 (0.28-0.35) | | -7.9 | 0.08 | | 0.24 (0.22-0.27) | | **-29.4** | **<.0001** | |
|  | **V** | 0.56 (0.53-0.62) | | -6.6 | 0.46 | | 0.49 (0.42-0-53) | | **-18.3** | **0.002** | |
| **Superior** | **VI** | 1.21 (1.16-1.26) | | -11.0 | 0.07 | | 0.94 (0.84-1.1) | | **-31.6** | **<.0001** | |
| **Posterior** | **Crus I** | 1.86 (1.61-2.04) | | -2.1 | 0.68 | | 1.60 (1.44-1.72) | | **-15.7** | **0.007** | |
|  | **Crus II** | 1.25 (1.04-1.35) | | 2.4 | 0.96 | | 0.98 (0.86-1.16) | | **-19.8** | **0.005** | |
|  | **VIIB** | 0.01 (0.61-0.76) | | 0 | ~~-~~ | | 0.55 (0.49-0.62) | | **-21.4** | **0.0002** | |
| **Inferior** | **VIIIA** | 0.87 (0.77-0.91) | | -3.1 | 0.38 | | 0.68 (0.64-0.82) | | **-23.5** | **0.001** | |
| **Posterior** | **VIIIB** | 0.57 (0.52-0.61) | | 0.5 | 0.27 | | 0.45 (0.40-0.54) | | **-22.8** | **0.005** | |
|  | **IX** | 0.52 (0.48-0.64) | | -4.9 | 0.46 | | 0.44 (0.40-0.57) | | -13.7 | 0.14 | |
| **Flocculo-nodular** | **X** | 0.09 (0.84-0.10) | | 11.7 | 0.96 | | 0.08 (0.07-0.09) | | **-10.1** | **0.005** | |
|  |  |  | |  |  | |  | |  |  | |

* Difference with controls, the following formula was used: [(Median _Participant group_ - median_CTR_) / median_CTR_] ∙ 100, where the participant group was pre-SCA2 or SCA2.

^§^exact p-value: the Wilcoxon rank-sum test was used to detect participant groups effect respect to CTR. No correction for multiple comparisons was applied.

**Supplementary Table 1b. Baseline sMRI cerebellar lobule thickness in SCA2 gene mutation carriers compared with controls.**

|  | | |  | **SCA2 presymptomatic subjects (n.13)** | | |  |  | **SCA2 Patients (n.14)** | | |  |
| --- | --- | --- | --- | --- | --- | --- | --- | --- | --- | --- | --- | --- |
|  | **Cerebellar lobule Thickness^#^** | | **Median**  **(Q1-Q3)** | | ** with**  **CTR^*^** | **p value^°°^** | | **Median**  **(Q1-Q3)** | | ** with CTR^*^** | **p value^°°^** | |
| **Anterior** | | **I-II** | 1.77 (1.56-2.14) | | 12.7 | 0.06 | | 1.22 (0.84-1.33) | | **-22.3** | **0.0007** | |
|  | | **III** | 3.56 (3.41-3.78) | | 5.6 | 0.06 | | 2.91 (2.62-3.22) | | **-13.6** | **0.003** | |
|  | | **IV** | 4.94 (4.91-5.06) | | 0.4 | 0.53 | | 4.57 (4.37-4.94) | | **-7.1** | **0.010** | |
|  | | **V** | 4.95 (4.84-5.17) | | 0.2 | 0.78 | | 4.47 (4.16-5.04) | | **-9.5** | **0.002** | |
| **Superior** | | **VI** | 5.02 (4.91-5.09) | | 0 | - | | 4.4 (3.76-4.71) | | **-12.2** | **<.0001** | |
| **Posterior** | | **Crus I** | 4.58 (4.38-4.76) | | - 0.86 | 0.73 | | 3.94 (3.72-4.19) | | **-14.7** | **<.0001** | |
|  | | **Crus II** | 4.31 (3.95-4.49) | | 1.6 | 0.52 | | 3.53 (3.34-4.05) | | **-19.4** | **0.0003** | |
|  | | **VIIB** | 4.61 (4.37-4.80) | | -2.74 | 0.11 | | 4.25 (4.07-4.39) | | **-10.3** | **<.0001** | |
| **Inferior** | | **VIIIA** | 4.67 (4.53-4.77) | | -2.3 | 0.11 | | 4.23 (3.97-4.55) | | **-11.5** | **0.0003** | |
| **Posterior** | | **VIIIB** | 4.60 (4.40-4.77) | | - 4.2 | 0.22 | | 3.49 (2.96-4.15) | | **-27.3** | **<.0001** | |
|  | | **IX** | 3.78 (3.43-4.11) | | - 1.8 | 0.25 | | 2.63 (2.26-2.98) | | **-31.6** | **<.0001** | |
| **Flocculo-nodular** | | **X** | 2.70 (2.28-2.97) | | 0.7 | 0.59 | | 1.13 (0.80-1.41) | | **-57.8** | **<.0001** | |

* Difference with controls, the following formula was used: [(Median _Participant group_ - median_CTR_) / median_CTR_] ∙ 100, where the participant group was pre-SCA2 or SCA2.

^§^exact p-value: the Wilcoxon rank-sum test was used to detect participant groups effect respect to CTR. No correction for multiple comparisons was applied.

**Supplementary Table 2a. Longitudinal sMRI changes in cerebellar lobule volume at 1-year follow-up, in patients and presymptomatic SCA2 subjects**

|  | |  | **SCA2 presymptomatic subjects (n.9)** | | |  |  | **SCA2 Patients (n.14)** | | |  |
| --- | --- | --- | --- | --- | --- | --- | --- | --- | --- | --- | --- |
| **Cerebellar lobule**  **Volume^#^** | | **Median**  **(Q1-Q3)** | | ** **  **with baseline^*^** | **p value^°°^** | | **Median**  **(Q1-Q3)** | | ****  **With baseline^*^** | **p value^°°^** | |
| **Anterior** | **I-II** | 0.008 (0.007 - 0.01) | | -3.5 | 0.51 | | 0.005 (0.004-0.007) | | 0 | - | |
|  | **III** | 0.10 (0.09-0.12) | | 0 | - | | 0.08 (0.06-0.09) | | 0 | - | |
|  | **IV** | 0.32 (0.29-0.34) | | 3.5 | 0.004 | | 0.24 (0.21-0.26) | | 0 | - | |
|  | **V** | 0.55 (0.53-0.65) | | -3.6 | 0.09 | | 0.47 (0.42-0-52) | | **-6.5** | **0.011** | |
| **Superior** | **VI** | 1.20 (1.17-1.38) | | -1.6 | 0.09 | | 0.91 (0.83-1.1) | | -3.3 | 0.07 | |
| **Posterior** | **Crus I** | 1.90 (1.82-2.05) | | 1.1 | 0.82 | | 1.59 (1.37-1.74) | | **-1.3** | **0.029** | |
|  | **Crus II** | 1.25 (1.08-1.34) | | 0 | - | | 0.95 (0.85-1.18) | | -3.1 | 0.32 | |
|  | **VIIB** | 0.71 (0.62-0.77) | | 0 | - | | 0.57 (0.48-0.61) | | 0 | - | |
| **Inferior** | **VIIIA** | 0.86 (0.83-0.91) | | -1.4 | 1.0 | | 0.66 (0.61-0.83) | | -3.0 | 0.06 | |
| **Posterior** | **VIIIB** | 0.58 (0.53-0.61) | | 0 | - | | 0.44 (0.39-0.51) | | 0 | - | |
|  | **IX** | 0.59 (0.47-0.67) | | -2.3 | 0.48 | | 0.44 (0.40-0.57) | | 0 | - | |
| **Flocculo-nodular** | **X** | 0.09 (0.86-0.10) | | 0 | - | | 0.08 (0.07-0.09) | | 1.2 | 0.34 | |
|  |  |  | |  |  | |  | |  |  | |

^#^Values are expressed as % of Total Intracranial Volume. **^*^**Difference for baseline**,**the following formula was used: [(Median_1-year follow-up_- median_baseline_) / median_baseline_] ∙ 100.

**^°°^**p-value: the Wilcoxon signed-rank test was used to detect time effect between baseline and 1-year follow-up evaluations. No correction for multiple comparisons was applied.

**Supplementary Table 2b. Longitudinal sMRI changes in cerebellar lobule thickness at 1-year follow-up, in patients and presymptomatic SCA2 subjects**

|  | |  | **SCA2 presymptomatic subjects (n.9)** | | |  |  | **SCA2 Patients (n.14)** | | |  |
| --- | --- | --- | --- | --- | --- | --- | --- | --- | --- | --- | --- |
| **Cerebellar lobule**  **Thickness^#^** | | **Median**  **(Q1-Q3)** | | ** **  **with baseline^*^** | **p value^°°^** | | **Median**  **(Q1-Q3)** | | ****  **With baseline^*^** | **p value^°°^** | |
| **Anterior** | **I-II** | 1.76 (1.45-1.89) | | -0.5 | 0.49 | | 1.12 (0.93-1.48) | | -8.1 | 0.96 | |
| **lobe** | **III** | 3.48 (3.31-3.72) | | -1.1 | 0.91 | | 2.77 (2.56-3.21) | | -4.8 | -0.15 | |
|  | **IV** | 5.03 (4.87-5.08) | | 1.8 | 0.55 | | 4.51 (4.33-4.91) | | **-1.3** | **0.025** | |
|  | **V** | 4.94 (04.88-5.12) | | -0.2 | 0.41 | | 4.43 (4.07-4.87) | | -0.9 | 0.09 | |
| **Superior** | **VI** | 5.07 (4.98-5.18) | | 0.2 | 0.09 | | 4.35 (4.03-4.70) | | -1.1 | 0.34 | |
| **Posterior** | **Crus I** | 4.69 (4.51-4.99) | | 0.8 | 0.29 | | 4.20 (3.75-4.24) | | 6.5 | 0.06 | |
|  | **Crus II** | 4.40 (4.28-4.83) | | -0.5 | 0.07 | | 3.90 (3.51-4.11) | | 10.4 | 0.17 | |
|  | **VIIB** | 4.76 (4.71-4.93) | | 3.0 | 0.01 | | 4.22 (4.08-4.45) | | -0.7 | 0.96 | |
| **Inferior** | **VIIIA** | 4.70 (4.64-4.81) | | 0.6 | 0.38 | | 4.43 (3.82-4.65) | | 4.7 | 0.89 | |
| **Posterior** | **VIIIB** | 4.79 (4.47-4.83) | | 0.4 | 0.33 | | 3.54 (2.96-4.16) | | 1.4 | 0.79 | |
|  | **IX** | 4.07 (3.85-4.25) | | 1.5 | 0.16 | | 2.52 (2.34-2.89) | | -4.2 | 0.81 | |
| **Flocculo-nodular** | **X** | 2.73 (2.48-2.97) | | 0.4 | 0.54 | | 0.92 (0.79-1.30) | | -18.5 | 0.33 | |
|  |  |  | |  |  | |  | |  |  | |

* Difference for baseline**,**the following formula was used: [(Median_1-year follow-up_- median_baseline_) / median_baseline_] ∙ 100.

**^°°^**p-value: the one-tailed Wilcoxon signed-rank test was used to detect time effect between baseline and 1-year follow-up evaluations. No correction for multiple comparisons was applied.

**Supplementary table 3. Evolution of SARA, SDMT, total cerebellar volume and mean cerebellar cortical thickness in SCA2 gene carriers**

|  | **SARA** | **SDMT** | **Pons volume**  **(%TIV)** | **Total Cerebellar Volume**  **(%TIV)** | **Mean Cerebellar cortical thickness** |
| --- | --- | --- | --- | --- | --- |
| Time to onset # | n. 13 preSCA2  n. 14 SCA2 | n. 13 preSCA2  n. 14 SCA2 | n. 13 preSCA2  n. 14 SCA2 | n. 13 preSCA2  n. 14 SCA2 | n. 13 preSCA2  n. 14 SCA2 |
| **Intercept** | 3.75 (0.748)  **p<.00001** | 42.86( 2.558)  **p<0.0001** | 0.64 (0.0251)  **p<0.0001** | 8.19 (0.201)  **p<0.0001** | 4.19 (0.046)  **p<0.0001** |
| **Time (per years)** | 0.36 (0.006)  **p<0.0001** | -0.598 (0.144)  **p=0.0004** | -0.0133 (0.0014)  **p<0.0001** | -0.086 (0.011)  **p<0.0001** | -0.027 (0.0026)  **p<0.0001** |
| **Q Time (per years)** | 0.0018 (0.003)  **p<0.0001** | -0.025 (0.011)  **p=0.036** | -0.000016 (0.00011)  p=0.157 | -0.002 (0.0009)  **p=0.022** | -0.00029 (0.00021)  p=0.18 |
| **Cubic Time (per year)** | 0.00027 (0.00018)  p= 0.158 | - | - | - | - |

#years from ataxia onset and enrollment in the study for symptomatic SCA2 patient, and expected years to onset for presymptomatic subjects, calculated on the basis of CAG length. In parenthesis Standard Error Mean.

SARA: Scale for Assessment and Rating of Ataxia; SDMT: Symbol Digit Modalities Test;TIV: Total Intracranial Volume

**Supplementary table 4. MRI volumetric analyses at baseline and 1-year follow-up in CTR, preSCA2 and SCA2 patients**

| **Subcortical brain structures**  **(%of TIV)** | **Subjects*** | **Descriptive statistics**  **Median (Q1-Q3)** | | | | **P-value°°** | |
| --- | --- | --- | --- | --- | --- | --- | --- |
|  |  | **Baseline** | **1-year follow-up** | | | **Comparison**  **with CTR** | |
| **Total Cerebellum Volume** | CTR | 9.59 (8.80-9.92) | | 9.59 (8.81-9.97) | | | - |
|  | PreSCA2 | 8.94 (8.63-9.59) | | 9.04 (8.76-9.60) | | | 0.51 |
|  | SCA2 | 7.47 (6.88-7.85) | | 7.32 (6.79-7.74) | | | **<0.0001** |
|  |  |  | |  | | |  |
| **Brain Medulla** | CTR | 0.29 (0.27-0.34) | | 0.29 (0.27-0.33) | | | - |
|  | PreSCA2 | 0.28 (0.26-0.30) | | 0.29 (0.28-0.30) | | | 0.91 |
|  | SCA2 | 0.23 (0.22-0.24) | | 0.23 (0.22-0.24) | | | **<0.0001** |
|  |  |  | |  | | |  |
| **Pons** | CTR | 0.93 (0.82-1.02) | | 0.95 (0.84-1.03) | | | - |
|  | PreSCA2 | 0.84 (0.77-0.89) | | 0.90 (0.83-0.92) | | | 0.049 |
|  | SCA2 | 0.47 (0.44-0.54) | | 0.44 (0.40-0.52) | | | **<0.0001** |
|  |  |  | |  | | |  |
| **Superior Cerebellar Peduncles** | CTR | 0.015 (0.013-0.017) | | 0.015 (0.013-0.017) | | | - |
|  | PreSCA2 | 0.013 (0.012-0.015) | | 0.014 (0.013-0.015) | | | 0.19 |
|  | SCA2 | 0.008 (0.008-0.010) | | 0.007 (0.007-0.009) | | | **<0.0001** |
|  |  |  | |  | | |  |
| **Midbrain** | CTR | 0.34 (0.32-0.39) | | | 0.39 (0.35-0.41) | | - |
|  | PreSCA2 | 0.35 (0.34-0.37) | | | 0.37 (0.36-0.39) | | 0.55 |
|  | SCA2 | 0.34 (0.31-0.34) | | | 0.30 (0.29-0.33) | | **<0.0001** |
|  |  |  | | |  | |  |
| **Thalamus** | CTR | 0.48 (0.47-0.53) | | | 0.50 (0.47-0.53) | | - |
|  | PreSCA2 | 0.49 (0.48-0.54) | | | 0.50 (0.50-0.51) | | 0.63 |
|  | SCA2 | 0.45 (0.41-0.46) | | | 0.43 (0.42-0.46) | | **0.0002** |
|  |  |  | | |  | |  |
| **Caudate** | CTR | 0.22 (0.21-0.24) | | | 0.22 (0.20-0.24) | | - |
|  | PreSCA2 | 0.21 (0.19-0.22) | | | 0.20 (0.19-0.22) | | 0.73 |
|  | SCA2 | 0.20 (0.19-0.22) | | | 0.19 (0.19-0.22) | | 0.07 |
|  |  |  | | |  | |  |
| **Putamen** | CTR | 0.30 (0.29-0.31) | | | 0.30 (0.29-0.31) | | - |
|  | PreSCA2 | 0.30 (0.28-0.32) | | | 0.30 (0.27-0.31) | | 0.95 |
|  | SCA2 | 0.27 (0.26-0.29) | | | 0.27 (0.25-0.28) | | **0.020** |
|  |  |  | | |  | |  |
| **Pallidum** | CTR | 0.13 (0.12-0.14) | | | 0.12 (0.12-0.13) | | - |
|  | PreSCA2 | 0.12 (0.12-0.13) | | | 0.12 (0.11-0.13) | | 0.15 |
|  | SCA2 | 0.11 (0.10-0.12) | | | 0.12 (0.10-0.13) | | **0.007** |
|  |  |  | | |  | |  |
| **Corpus callosum** | CTR | 0.27 (0.25-0.31) | | | 0.27 (0.24-0.29) | | - |
|  | PreSCA2 | 0.25 (0.24-0.29) | | | 0.25 (0.23-0.28) | | 0.28 |
|  | SCA2 | 0.22 (0.21-0.25) | | | 0.23 (0.20-0.26) | | **0.006** |

*Participant groups: CTR: Controls (n =15); PreSCA2: presymptomatic SCA2 (n =9); SCA2 affected patients (n =14). TIV= Total intracranial volume. °°p-value: the Wilcoxon rank-sum test was used to detect differences between participant groups
